# MRI Diffusion Imaging as an Additional Biomarker for Monitoring Chemotherapy Efficacy in Tumors

**DOI:** 10.3390/medicina62010173

**Published:** 2026-01-15

**Authors:** Małgorzata Grzywińska, Anna Sobolewska, Małgorzata Krawczyk, Ewa Wierzchosławska, Dominik Świętoń

**Affiliations:** 1 Neuroinformatics and Artificial Intelligence Lab, Department of Neurophysiology, Neuropsychology and Neuroinformatics, Medical University of Gdansk, 80-210 Gdansk, Poland; 2 Department of Radiology, University Clinical Center, 80-952 Gdansk, Poland; 3 2nd Department of Pediatrics, Haemathology & Oncology, Medical University of Gdansk, 80-210 Gdansk, Poland; 4 Department of Radiology, Greater Poland Cancer Centre, 61-866 Poznań, Poland; 5 2nd Department of Radiology, Medical University of Gdansk, 80-210 Gdansk, Poland

**Keywords:** rhabdomyosarcoma, soft tissue sarcoma, magnetic resonance imaging, diffusion-weighted imaging, apparent diffusion coefficient, pediatric oncology

## Abstract

*Background and Objectives*: Soft tissue sarcomas account for approximately 7% of all malignant tumors in the pediatric population. Diffusion-weighted imaging (DWI) with apparent diffusion coefficient (ADC) measurements may provide early functional biomarkers of treatment response by reflecting changes in tumor cellularity. This study evaluated whether ADC-derived parameters can serve as quantitative biomarkers of neoadjuvant chemotherapy response in pediatric rhabdomyosarcoma. *Materials and Methods*: This retrospective single-center study included 14 patients aged ≤18 years with histopathologically confirmed rhabdomyosarcoma who underwent MRI before treatment and after three cycles of chemotherapy. Twenty-five patients were initially identified; eleven were excluded due to imaging artifacts or absence of baseline examination. ADC maps were generated on 1.5T and 3T scanners. Regions of interest were placed over the entire lesion and areas with the lowest ADC signal. Relative ADC (rADC) was calculated by normalizing tumor ADC to adjacent healthy muscle. Paired *t*-tests were used to compare pre- and post-treatment values. *Results*: At baseline, 13/14 patients (93%) demonstrated diffusion restriction. Mean ADC increased from 1.11 × 10^−3^ mm^2^/s (SD ± 0.48) at baseline to 1.63 × 10^−3^ mm^2^/s (SD ± 0.67) after treatment. The paired t-test for rADC yielded t = −3.089 (*p* = 0.0086, 95% CI: −0.79 to −0.14), indicating a statistically significant change. There was a significant difference between the ADC values of the entire lesion and the areas with the lowest signal in tumors with a heterogenic structure, t = 2.862, *p* = 0.013. *Conclusions*: ADC and rADC increased significantly after neoadjuvant chemotherapy in pediatric rhabdomyosarcoma, suggesting potential utility as early functional biomarkers of treatment response. These preliminary findings require validation in larger multicenter prospective studies with correlation to histopathological response and clinical outcomes before clinical implementation.

## 1. Introduction

Soft tissue sarcomas make up about 7% of all malignant tumors in the pediatric population [1,2,3], and present diagnostic and therapeutic challenges due to their heterogeneous presentation and variable clinical symptoms depending on tumor location and cellular structure [4,5]. Magnetic Resonance Imaging (MRI) plays an essential role in pediatric soft tissue sarcoma management, providing superior soft tissue contrast and precise anatomical delineation essential for surgical planning and treatment monitoring [3,6]. MRI excels at providing detailed morphological images and is often complemented by computed tomography (CT) for evaluating bone involvement and distant metastases [7], and positron emission tomography (PET) for assessing metabolic activity and treatment response [4]. MRI excels at providing detailed images of soft tissue contrast and defining the extent of the tumor, which is vital for surgical planning and assessing the feasibility of complete resection [3]. However, MRI results are often complemented by other imaging modalities, such as computed tomography (CT) and positron emission tomography (PET) scans. CT scans are beneficial for evaluating bone involvement and detecting distant metastases [7]. In contrast, PET scans help assess tumor metabolic activity, which can indicate tumor aggressiveness or response to treatment [4,8].

Beyond imaging, biopsy and histopathological analysis remains the gold standard for definitive diagnosis and molecular characterization [8,9,10].

The integration of these diagnostic modalities enables multidisciplinary teams to formulate optimal treatment strategies and monitor therapeutic response [1,4,11,12,13]. MRI is crucial for diagnosing and managing soft tissue sarcomas, particularly diffusion-weighted imaging (DWI), offering insight into tumor microstructure beyond conventional anatomical imaging [1,14]. As imaging technology has evolved, multiparametric MRI has become increasingly significant.

**Figure 1:**
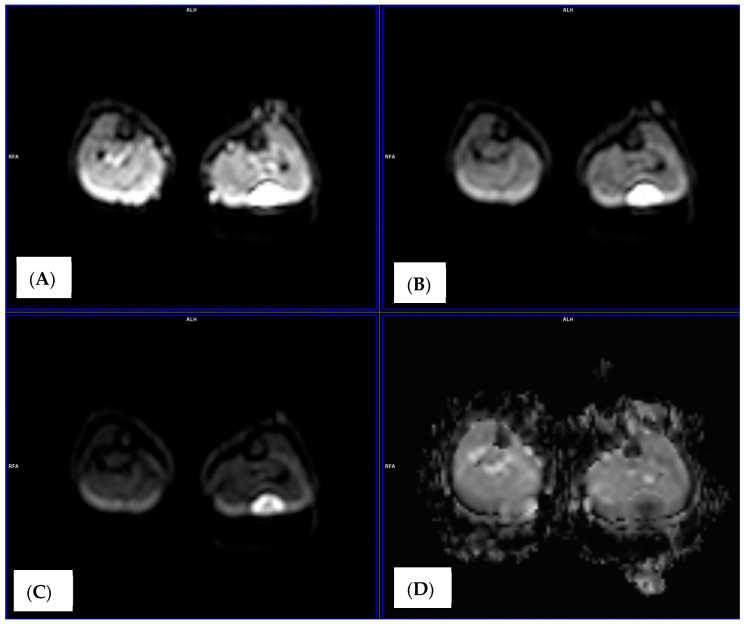
Transverse RMS image of the left lower leg in a pediatric patient in diffusion images (DWI): (**A**) DWI b = 0, (**B**) DWI b = 300, (**C**) DWI b = 900, (**D**) apparent diffusion coefficient (ADC) map.

DWI quantifies water molecule diffusion within tissues, generating apparent diffusion coefficient (ADC) maps that reflect tissue cellularity and microarchitecture [15] (Figure 1).

High cellular density restricts diffusion, resulting in lower ADC values characteristic of malignancy. Effective cytotoxic therapy reduces cellularity and increases extracellular space, resulting in elevated ADC values. This relationship enables DWI to serve as an early functional biomarker of treatment response, potentially preceding morphological changes detectable by size-based criteria (Figure 1). On the other hand, perfusion imaging assesses the blood flow within the tumor, providing insights into its metabolic demands and vascular supply. Tumors with high perfusion rates may be more aggressive or respond differently to specific therapies.

When used together, these imaging techniques enhance the clinician’s ability to monitor tumor response to treatment over time. Thus, multiparametric MRI not only aids in distinguishing malignant from benign lesions but also plays a pivotal role in evaluating the efficacy of the chosen treatment regimen, helping tailor therapies to individual patient needs more effectively.

Despite its potential utility, the literature on ADC as a response biomarker in pediatric rhabdomyosarcoma remains limited. This study aimed to evaluate whether ADC values from diffusion-weighted sequences can serve as quantitative biomarkers for assessing chemotherapy response in this population.

## 2. Materials and Methods

### 2.1. Study Design and Ethical Approval

The Independent Bioethics Committee for Scientific Research at the Medical University of Gdańsk provided ethical approval for this study (NKBBN/223/2022). The study group includes patients treated at the Department of Pediatrics, Hematology, and Oncology at the University Clinical Center in Gdańsk between 2012 and 2022, who underwent Magnetic Resonance Imaging (MRI) before starting treatment and after three blocks of induction chemotherapy, according to CWS 2006.

### 2.2. Patient Population

Patients up to 18 years of age with a histopathologically confirmed diagnosis of rhabdomyosarcoma were included. The group of children diagnosed with RMS was initially 25. Still, after data analysis, this number decreased to 14 due to magnetic susceptibility artifacts or motion artifacts in the DWI sequence and the absence of baseline MRI examination before the commencement of treatment.

### 2.3. MRI Acquisition

The scans were performed at the Department of Radiology, University Clinical Center in Gdańsk using Magnetom Aera 1.5 T (Siemens, Erlangen, Germany), Magnetom Sola 1.5T (Siemens, Erlangen, Germany) and Achieva 3T TX (Philips, Eindhoven, The Netherlands). Imaging was conducted using standard protocols tailored to the region examined, including diffusion sequence and anatomical sequences before and after intravenous administration of gadolinium contrast agent.

### 2.4. Image Analysis and ADC Measurements

Magnetic Resonance Imaging (MRI) studies of patients were analyzed using Siemens software—Syngo.via 40 apparent diffusion coefficient (ADC) values. The software used in most MRI machines generates ADC maps automatically or semi-automatically. The calculated ADC value is independent of the magnetic field strength. A voxel on the ADC map describes the relationship between the logarithm of signal intensity and the b-value [12].

$$ADC=\frac{ln\left(\frac{SI}{{SI}_{o}}\right)}{b}$$

SI—signal intensity of the DWI image, SI_0_—signal intensity in the T2-weighted image for acquisition without magnetic field gradients with a b-value of 0.

Quantitative measurements of the signal on ADC maps were performed, where the ROI area covered the entire lesion (area of reduced diffusion, with an attempt to avoid the edges of the lesion and any surrounding fluid spaces that could falsify the result), as well as the ROI area of the lesion with the lowest signal.

To account for inter-scanner and protocol-related variability inherent in this retrospective study, we calculated relative ADC (rADC) by normalizing tumor ADC values to adjacent healthy muscle tissue within the same acquisition.

**Figure 2:**
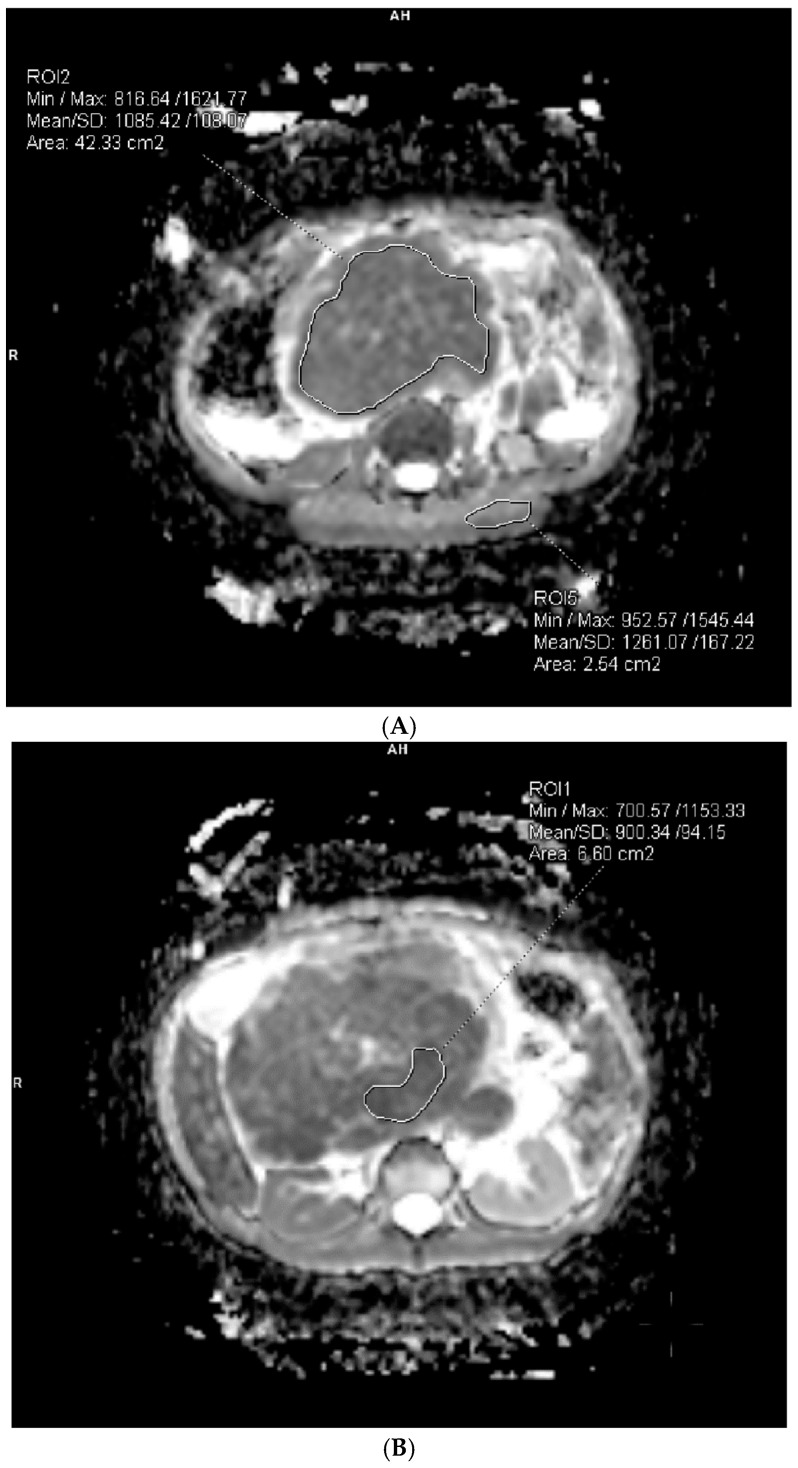
(**A**) Delineation of ROI for the entire RMS tumor area and ROI for the area of healthy striated skeletal muscle tissue. (**B**) Designation of ROI as the area with the lowest ADC values of the tumor.

The relative apparent diffusion coefficient (rADC) was defined as the ratio of the mean ADC value from the entire rhabdomyosarcoma lesion to the ADC of adjacent healthy muscle tissue (Figure 2).

$$rADC=\frac{{ADC}_{tumor}}{{ADC}_{muscle}}$$

### 2.5. Statistical Analysis

Statistical analyses were performed using the pandas’ library in Python v. 3.8.3 (McKinney, 2010). The statistical analysis used the mean signal value in the designated areas of interest (ROI). Descriptive statistics included mean, standard deviation (SD), median, and range. Paired *t*-tests were used to compare ADC and rADC values before and after chemotherapy at 95% CI. The percentage change in ADC was calculated as [(post-treatment ADC − pre-treatment ADC)/pre-treatment ADC] × 100%. Statistical significance was set at *p* < 0.05. Some of the studies of one patient were performed on different MRI machines; therefore, as a reference point, the signal in ROI areas in healthy striated muscles was also measured. An experienced radiology specialist verified the ROI areas.

A power analysis was not performed a priori due to the retrospective nature of this exploratory study. Given the small sample size (n = 14), our results should be interpreted as preliminary and hypothesis-generating.

## 3. Results

### 3.1. Baseline Diffusion Characteristics

We observed decreased ADC values at the time of RMS tumor diagnosis in most pediatric patients 13/14 (92%). The average ADC value at the level of the largest diameter of the tumor, taken from the entire area, was 1.11 × 10^−3^ mm^2^/s; SD ± 0.48 × 10^−3^ mm^2^/s; value range 0.59–2.53 × 10^−3^ mm^2^/s, with a median of 1.09 × 10^−3^ mm^2^/s. The detailed values are presented in Table 1. The rADC values were also decreased at baseline, with a mean of 0.95 (SD ± 0.35; range 0.47–1.82; median 0.99). In the studied group, for nine patients whose tumors were large and had a heterogeneous structure before the start of treatment, the ADC values were averages from different tumor components. Therefore, quantitative assessments of the areas with the lowest signal on the ADC map were conducted. Measurements of the lowest ADC signal of the tumor were made only before the start of treatment in patients with distinct areas.

A paired *t*-test showed a significant difference between the ADC values of the entire lesion and the areas with the lowest signal in tumors with heterogenic structure, t = 2.862, *p* = 0.013. This result indicates a statistically significant difference between the ADC values of the entire lesion and those of the lowest-signal area. This could imply that different areas within the lesions have varying levels of diffusion, potentially reflecting heterogeneity in the tumor’s cellular environment or the presence of other pathological features (Table 1).

### 3.2. ADC Changes After Chemotherapy

After three cycles of chemotherapy, all patients showed a reduction in tumor size (partial response according to RECIST criteria) and a more homogeneous structure, allowing ADC values to be assessed across the entire residual tumor mass (Table 2).

Mean ADC values after treatment were 1.63 × 10^−3^ mm^2^/s; SD ± 0.67 × 10^−3^ mm^2^/s; range of values 0.68–3.34 × 10^−3^ mm^2^/s, while the median was 1.37 × 10^−3^ mm^2^/s. The absolute difference between ADC on baseline and after treatment was mean 0.52 (range −0.09 to 1.34) (Table 2). Interestingly, the difference between the initial study and after chemotherapy were similar to the absolute difference values of the ADC, mean 0.46 (range 0.51 to 1.76).

**Figure 3:**
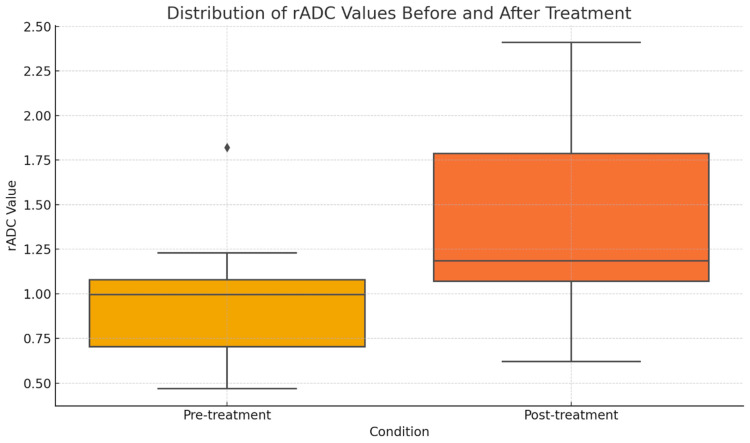
Distribution of rADC values before and after treatment. Diamond symbol (◆) indicates outlier.

Similarly post-treatment rADC values were significantly lower than before therapy, t = −3.089 (*p* = 0.0086, 95% CI: −0.79 to −0.14) (Figure 3).

Both findings demonstrated a difference in rADC values before and after treatment, suggesting the potential usefulness of this parameter in monitoring therapy response, as it is independent of imaging conditions.

**Figure 4:**
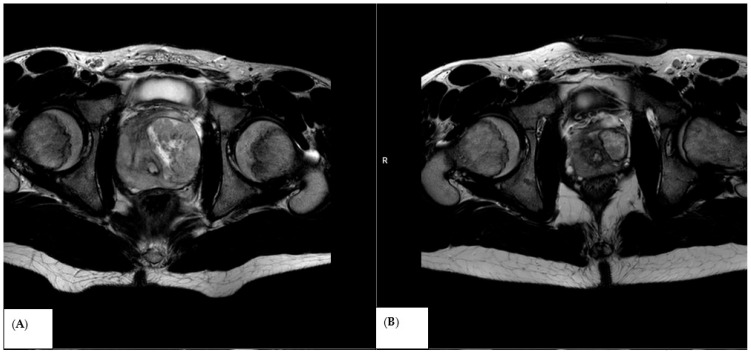
T2-weighted image in the transverse plane of the RMS prostate tumor, (**A**) before treatment and (**B**) after 3 cycles of chemotherapy.

**Figure 5:**
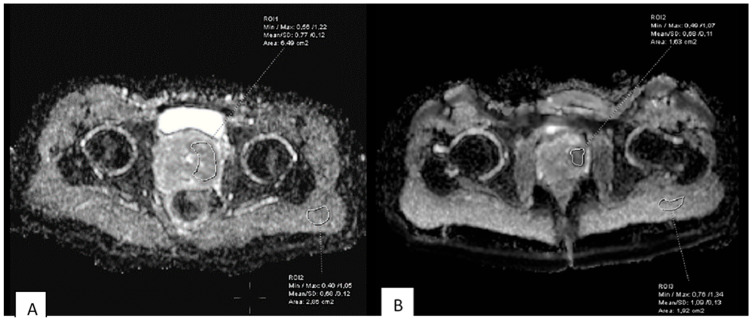
Image of the ADC map in the transverse plane of the RMS prostate tumor, (**A**) before treatment and (**B**) after the 3rd cycle of chemotherapy.

In three cases, the ADC values did not increase after treatment; however, the tumor significantly decreased in size (Figure 4 and Figure 5).

## 4. Discussion

Previous single studies have shown that significant increases in ADC values after treatment often correlate with better clinical outcomes and prognosis. Therefore, ADC values can be used to monitor treatment response and predict long-term patient outcomes. In this retrospective study of pediatric rhabdomyosarcoma, all except one case demonstrated diffusion restriction at diagnosis, consistent with high cellularity and limited extracellular space. The baseline mean ADC values in our cohort were 1.11 × 10^−3^ mm^2^/s; SD ± 0.48 × 10^−3^ mm^2^/s; value range 0.59–2.53 × 10^−3^ mm^2^/s, which is in agreement within previously reported ranges for soft tissue sarcomas. In the largest available multicenter study by van Ewijk et al. [16], the mean ADC value was 1.13 × 10^−3^ mm^2^/s, value range 0.7–1.2 × 10^−3^ mm^2^/s. Another previous systematic review showed the range of mean ADC value for rhabdomyosarcoma from 0.78 to 1.21 × 10^−3^ mm^2^/s [17], which is also in accordance with our results.

The most significant problem with ADC interpretation is the heterogenicity of the tumors influencing the ADC values, like necrosis, bleeding, or mucinous content. In our study, we also compared results of two ways of measuring, one with the ROI placed in the whole diameter of the tumor, and the another taking the areas of the lowest signal on the ADC map, avoiding cystic areas and bleeding. The difference was statistically significant; the paired t-test conducted on the ADC values before and after treatment yielded a *p*-value of 0.0086. The development of consensus guidelines for DWI acquisition and ADC analysis in pediatric sarcomas seems to be necessary [14,15,16,17].

The mean absolute ADC change after neoadjuvant chemotherapy was 0.52 (range −0.09 to 1.34) when assessed the around whole area of the tumor, which is in accordance with other authors [15]. A significant increase in ADC values post-treatment is generally associated with a positive response to therapy. It indicates that chemotherapy has effectively reduced the tumor burden by killing cancer cells and reducing cellularity. Conversely, if ADC values do not change significantly or decrease, it may suggest that the tumor is not responding well to the treatment, indicating resistance or ineffective chemotherapy. The authors compared the changes in the rADC value before and after treatment, which seems to be a promising parameter, overtaking changes in imaging parameters between exams; however, there is a need for further evaluation on a larger group of patients. By monitoring ADC/rADC values, clinicians can assess the effectiveness of therapies sooner than traditional imaging methods, allowing for timely adjustments and potentially improving patient outcomes.

Lower ADC values before treatment typically indicate high cellular density and restricted diffusion within the tumor. Densely packed tumor cells limit the movement of water molecules, resulting in lower ADC values. An increase in ADC values after treatment often suggests a reduction in tumor cellular density. Chemotherapy can induce cell death and necrosis, leading to the breakdown of cellular structures and an increase in extracellular space, which allows for greater free diffusion of water molecules. By monitoring ADC changes, clinicians can tailor treatment plans to individual patients. For instance, if a tumor demonstrates significant ADC increase early in treatment, it could support continuing the current therapy. Furthermore, DWI-based monitoring offers a radiation-free alternative to conventional imaging modalities such as CT or FDG PET/CT, which involve cumulative ionizing radiation exposure—a particular concern in pediatric populations requiring serial imaging. Due to the limited availability of concurrent PET/CT data in our cohort, we were unable to perform comparative analysis between metabolic and diffusion-based response parameters. FDG PET/CT provides complementary information on tumor metabolic activity and has established utility in initial staging, restaging, and prognostic stratification of soft tissue sarcomas [18]. Future prospective studies incorporating both DWI-derived ADC measurements and FDG PET/CT metabolic parameters (SUVmax, metabolic tumor volume) would enable systematic comparison of these modalities and potentially identify optimal multiparametric approaches for chemotherapy response assessment in pediatric rhabdomyosarcoma.

The significance of ADC changes lies in their ability to provide a non-invasive, early indication of how a tumor responds to treatment. This can greatly aid in making timely and informed clinical decisions, ultimately improving patient management and outcomes in pediatric soft tissue sarcomas. Despite the small study cohort, these findings may contribute to future multicenter meta-analyses.

### Limitations

First, this study is retrospective and single-center, with a relatively small final sample size (n = 14), which limits the generalizability of the findings.

Second, MRI examinations were performed on three different scanners (two 1.5T systems and one 3T system) with standard but not strictly standardized acquisition protocols. Although we employed rADC normalization to mitigate inter-scanner variability, this approach cannot fully compensate for differences in field strength, gradient systems, and sequence parameters.

Third, we did not directly correlate ADC changes with histopathological response assessment, risk group stratification, or long-term clinical outcomes such as event-free or overall survival.

Fourth, we did not assess inter- or intra-observer variability for ROI placement on ADC maps due to the small number of patients.

Future multicenter, prospective studies with larger pediatric cohorts and standardized DWI protocols are needed to confirm our findings and to establish ADC-based response criteria in rhabdomyosarcoma.

## 5. Conclusions

ADC and rADC derived from diffusion-weighted MRI significantly increased after neoadjuvant chemotherapy in pediatric rhabdomyosarcoma. These results support the use of quantitative diffusion parameters as non-invasive imaging biomarkers for assessing treatment response in pediatric soft tissue sarcomas. Larger multicenter prospective studies with standardized protocols and systematic correlation with histopathological response and survival outcomes are needed to validate these findings and establish clinically actionable ADC-based response criteria.

## Figures and Tables

**Table 1:** ADC and rADC values of analyzed tumors at baseline exam.

No.	Whole Area ADC Value (×10^−3^ mm^2^/s)	Whole Area rADC Value (×10^−3^ mm^2^/s)	Area with Lowest ADC Value (×10^−3^ mm^2^/s)	Area with Lowest rADC Value (×10^−3^ mm^2^/s)
1	1.09	0.86	0.90	0.71
2	1.19	1.23	1.19	1.23
3	0.77	0.65	0.77	0.65
4	0.72	0.58	0.71	0.57
5	1.10	1.04	0.89	0.85
6	0.94	1.01	0.54	0.58
7	1.58	1.08	1.58	1.08
8	0.77	1.13	0.77	0.94
9	0.61	0.47	0.61	0.47
10	0.59	0.47	0.54	0.43
11	1.23	0.98	0.93	0.74
12	1.34	1.07	1.34	1.07
13	1.09	0.96	0.99	0.87
14	2.53	1.82	2.10	1.51

ADC, apparent diffusion coefficient; rADC, relative apparent diffusion coefficient.

**Table 2:** ADC and rADC values from entire area and area with lowest signal before and after treatment.

No.	Whole Area ADC Value (×10^−3^ mm^2^/s)	ADC Change After Treatment (%)	Area with Lowest ADC Value (×10^−3^ mm^2^/s)	ADC Change After Treatment (%)	Whole Area rADC Value (×10^−3^ mm^2^/s)	rADC Change After Treatment (%)	Area with Lowest rADC Value (×10^−3^ mm^2^/s)	rADC Change After Treatment (%)
1	1.38	127	1.38	153	1.1	128	0.91	128
2	1.94	163	1.94	163	1.58	128	1.29	105
3	1.96	255	1.96	255	2.41	371	3.74	575
4	2.06	286	2.06	290	1.83	316	3.15	553
5	1.16	105	1.16	130	1.1	106	1.05	124
6	1.09	116	1.09	202	1.06	105	1.05	181
7	1.54	97	1.54	97	1.66	154	1.53	142
8	0.68	88	0.68	88	0.62	55	0.55	59
9	1.27	208	1.27	208	0.91	194	1.93	411
10	1.37	232	1.37	254	1.19	253	1.79	416
11	1.17	95	1.17	126	1.02	104	1.04	141
12	2.57	192	2.57	192	1.92	179	1.79	167
13	1.33	122	1.33	134	1.18	123	1.24	143
14	3.34	132	3.34	159	2.27	125	1.25	83

ADC, apparent diffusion coefficient; rADC, relative apparent diffusion coefficient.

## Data Availability

The data presented in this study are available on reasonable and qualified research request from the corresponding author. Data requestors will need to sign a data access agreement.
